# Functional Activities of O-Polysaccharide and Hemolysin Coregulated Protein 1 Specific Antibodies Isolated from Melioidosis Patients

**DOI:** 10.1128/iai.00214-22

**Published:** 2022-10-13

**Authors:** Apinya Pumpuang, Suporn Paksanont, Mary N. Burtnick, Paul J. Brett, Narisara Chantratita

**Affiliations:** a Department of Microbiology and Immunology, Faculty of Tropical Medicine, Mahidol University, Bangkok, Thailand; b Department of Clinical Pathology, Faculty of Medicine, Vajira Hospital, Navamindradhiraj University, Bangkok, Thailand; c Mahidol-Oxford Tropical Medicine Research Unit, Faculty of Tropical Medicine, Mahidol University, Bangkok, Thailand; d Department of Microbiology and Immunology, University of Nevada, Reno School of Medicine, Reno, Nevada, USA; Stanford University

**Keywords:** *Burkholderia pseudomallei*, O-polysaccharide, Hcp1, antibody function, ADCP, ADCD, vaccine, melioidosis, antigen, opsonization

## Abstract

Melioidosis is a fatal tropical disease caused by the environmental Gram-negative bacterium, Burkholderia pseudomallei. This bacterium is intrinsically resistant to several antibiotics and treatment of melioidosis requires prolonged antibiotic administration. To date, there are no vaccines available for melioidosis. Previous studies have shown that humoral immunity is critical for surviving melioidosis and that O-polysaccharide (OPS) and hemolysin coregulated protein 1 (Hcp1) are important protective antigens in animal models of melioidosis. Our previous studies revealed that melioidosis patients had high levels of OPS- and Hcp1-specific antibodies and that IgG against OPS (IgG-OPS) and Hcp1 (IgG-Hcp1) were associated with patient survival. In this study, we characterized the potential function(s) of IgG-OPS and IgG-Hcp1 from melioidosis patients. IgG-OPS and IgG-Hcp1 were purified from pooled serum obtained from melioidosis patients using immuno-affinity chromatography. Antibody-dependent cellular phagocytosis assays were performed with pooled serum from melioidosis patients and compared with serum obtained from healthy controls. Serum from melioidosis patients significantly enhanced B. pseudomallei uptake into the human monocytic cell line THP-1 compared with pooled serum from healthy donors. Enhanced opsonization was observed with IgG-OPS and IgG-Hcp1 in a dose-dependent manner. Antibody-dependent complement deposition assays were performed with IgG-OPS and IgG-Hcp1 using flow cytometry and showed that there was enhanced C3b deposition on the surface of B. pseudomallei treated with IgG-OPS but to a lesser degree with IgG-Hcp1. This study provides insight into the function of IgG-OPS and IgG-Hcp1 in human melioidosis and supports that OPS and Hcp1 are potential vaccine antigens for immunization against melioidosis.

## INTRODUCTION

Melioidosis, caused by the Gram-negative bacterium Burkholderia pseudomallei, is commonly associated with community acquired septicemia in regions of endemicity. The disease is typically found in tropical countries and is especially prevalent in northeast Thailand and northern Australia (1, 2). Worldwide, there is estimated to be 165,000 cases of melioidosis per year and 89,000 deaths (3). Clinical manifestations of melioidosis range from mild infections to severe sepsis. The majority of melioidosis patients present with bacteremia and pneumonia. In northeast Thailand and Southeast Asia, mortality rates can be as high as 40 to 50% (4, 5).

B. pseudomallei is an encapsulated bacterium and is intrinsically resistant to several antibiotics. Prolonged regimens that include intravenous antibiotics such as ceftazidime or carbarpenems followed by oral trimethroprim-co-trimoxazole are required for the treatment of melioidosis. Although, vaccines that stimulate capsule-specific antibody responses are effective for other encapsulated bacteria such as Streptococcus pneumoniae, Haemophilus influenzae type b, and *Neisseria* meningitidis (6–8), there are currently no vaccines available for protection against melioidosis. Therefore, there is a need to identify potential antigens for vaccine development.

O-polysaccharide (OPS) and hemolysin coregulated protein 1 (Hcp1) are important virulence factors expressed by B. pseudomallei and are considered to be promising vaccine candidates (9–11). OPS is a component of lipopolysaccharide (LPS) located on outer membrane of bacteria. Hcp1 is a protein component of the cluster 1 type VI secretion system (T6SS) that plays a role in the intracellular lifestyle of B. pseudomallei (9, 12–14). Both OPS and Hcp1 are recognized by the immune systems of melioidosis patients (15). Our previous studies demonstrated by ELISAs that melioidosis patients produced high levels of IgG against OPS and Hcp1 antigens (16, 17). OPS induced high levels of IgG1 and IgG2 subclasses while Hcp1 predominantly induced high levels of IgG1 (18).

Many studies in animal models have demonstrated the association between antibody levels and protection from melioidosis but the mechanisms of protection have not been well investigated (10, 19–22). A study in human melioidosis showed a lower mortality rate was associated with seropositivity against crude B. pseudomallei antigen preparations (23). We previously reported an association of survival with high levels of IgG against OPS and Hcp1 in melioidosis patients (18, 24) suggesting a potential functional role for these antibodies in protection against disease (25). In addition, Chaichana et al. demonstrated that serum from survivors of melioidosis enhanced bacterial uptake compared to serum from nonsurvivors (25). The same study demonstrated that purified IgG against whole-cell antigen promotes antibody-dependent cellular phagocytosis (ADCP). However, this study did not characterize what antigen-specific antibodies in the patient serum samples were associated with the ADCP (26).

We hypothesized that specific IgG antibodies against OPS (IgG-OPS) and Hcp1 (IgG-Hcp1) in human melioidosis cases could contribute to enhanced phagocytosis and complement deposition on B. pseudomallei. The aim of this study was to evaluate the roles of IgG-OPS and IgG-Hcp1 in ADCP and antibody-dependent complement deposition (ADCD) assays. We purified IgG antibodies from pooled serum obtained from melioidosis patients and healthy donors using immuno-affinity chromatography. We evaluated the ADCP activities of IgG-OPS and IgG-Hcp1 for their ability to promote bacterial uptake and survival in THP-1 cells. ADCD associated with IgG-OPS and IgG-Hcp1 was also determined by assessing C3b deposition on B. pseudomallei K96243 via flow cytometry. Results of these studies indicated that in both instances higher activity was associated with IgG-OPS compared to IgG-Hcp1.

## RESULTS

### Serum from human melioidosis patients enhances bacterial uptake into THP-1 cells.

**FIG 1:**
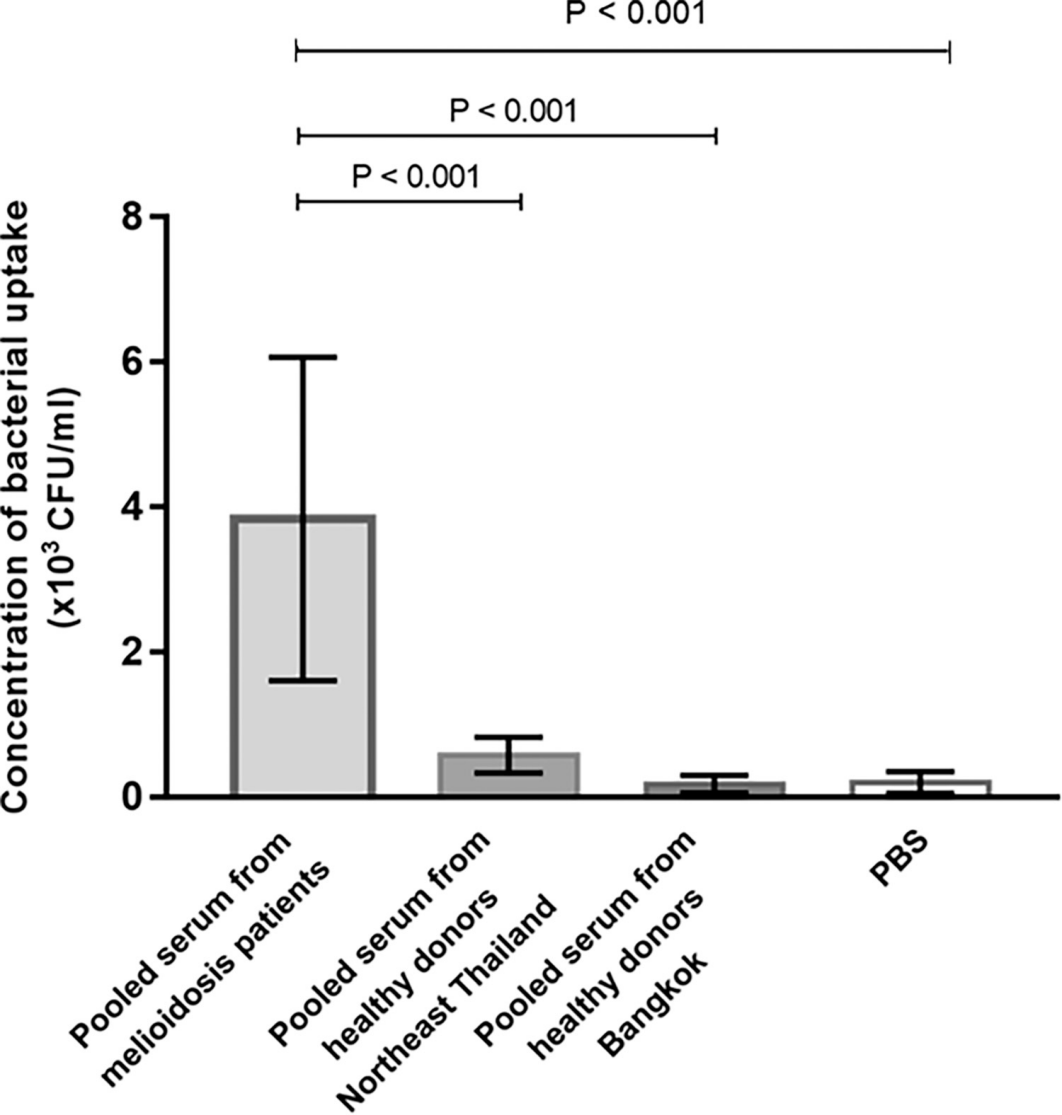
Antibody-dependent cellular phagocytosis (ADCP) activity from pooled serum from melioidosis patients compared to healthy donors from Northeast Thailand, healthy donors from Bangkok and PBS control in THP-1 cells. The ADCP activity was determined by colony count method to enumerate the live bacteria in THP-1 cells. Three-independent experiments were performed.

The opsonophagocytic activity of pooled serum from melioidosis patients and healthy controls was determined using THP-1 cells. We observed that pooled melioidosis serum at dilution of 1:10 significantly enhanced bacterial uptake into THP-1 cells compared with pooled serum from healthy donors from endemic and non-areas of endemicity. Results showed that mean concentrations ± standard deviation (SD) of bacterial uptake for pooled melioidosis serum was 3.83 ± 2.30× 10^3^ CFU/mL, pooled healthy donor serum from areas of endemicity was 0.58 ± 0.25× 10^3^ CFU/mL and pooled healthy donor serum from non-areas of endemicity was 0.18 ± 0.12× 10^3^ CFU/mL (Fig. 1). We examined live/dead THP1 cells using an inverted light microscope, but we did not observe the difference in live/dead cells among cells incubated with different serum groups and PBS control.

### Purification and analysis of IgG-OPS and IgG-Hcp1 antibodies.

**FIG 2:**
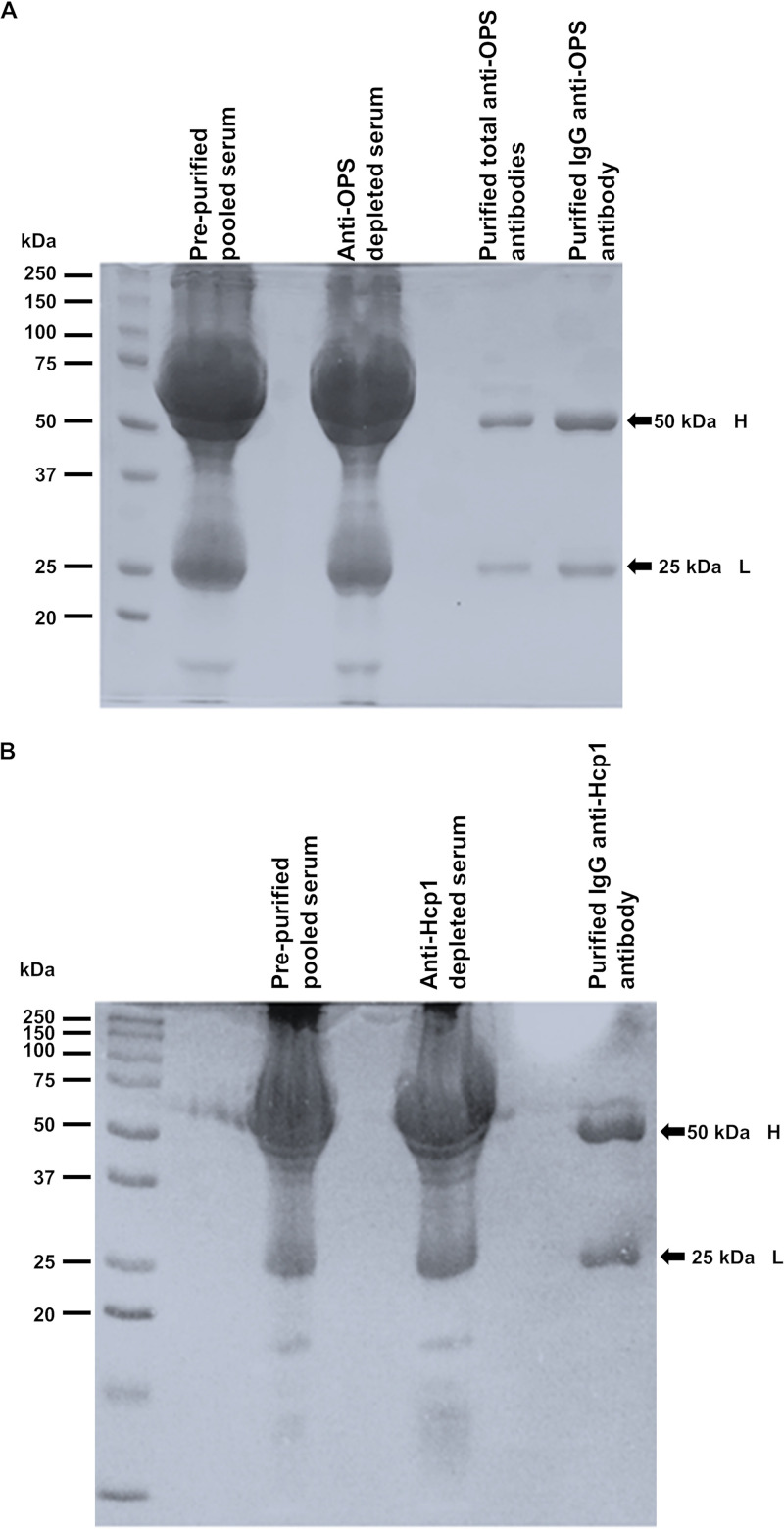
SDS-PAGE and Coomassie blue staining of prepurified pooled serum, depleted serum, purified anti-OPS antibodies, purified IgG anti-OPS and purified IgG anti-Hcp1. SDS-PAGE was performed on 14% gel. The separated proteins were stained with Coomassie blue. Size of proteins were compared to protein markers (kDa). Numbers on the left represent molecular weight markers. (A) Prepurified pooled serum, anti-OPS depleted serum, purified anti-OPS antibodies, and purified IgG anti-OPS. (B) Prepurified pooled serum, anti-Hcp1 depleted serum, and purified IgG anti-Hcp1. H, heavy chain; L, light chain.

IgG-OPS and IgG-Hcp1 samples were obtained from pooled melioidosis patient serum using a two-step approach. The total amount of IgG-OPS and IgG-Hcp1 isolated from 400 mL of the pooled patient serum (290 mL for IgG-OPS and 110 mL for IgG-Hcp1) was 3.92 mg and 6.25 mg, respectively. As expected, SDS-PAGE analysis of the pooled and depleted patient serum samples demonstrated a complex mixture of proteins. In contrast, the purified total Ig and IgG fractions revealed the presence of two major bands of ~50 kDa and ~25 kDa (Fig. 2). These observations are consistent with the molecular weights associated with IgG heavy and light chains in their reduced forms, respectively.

**FIG 3:**
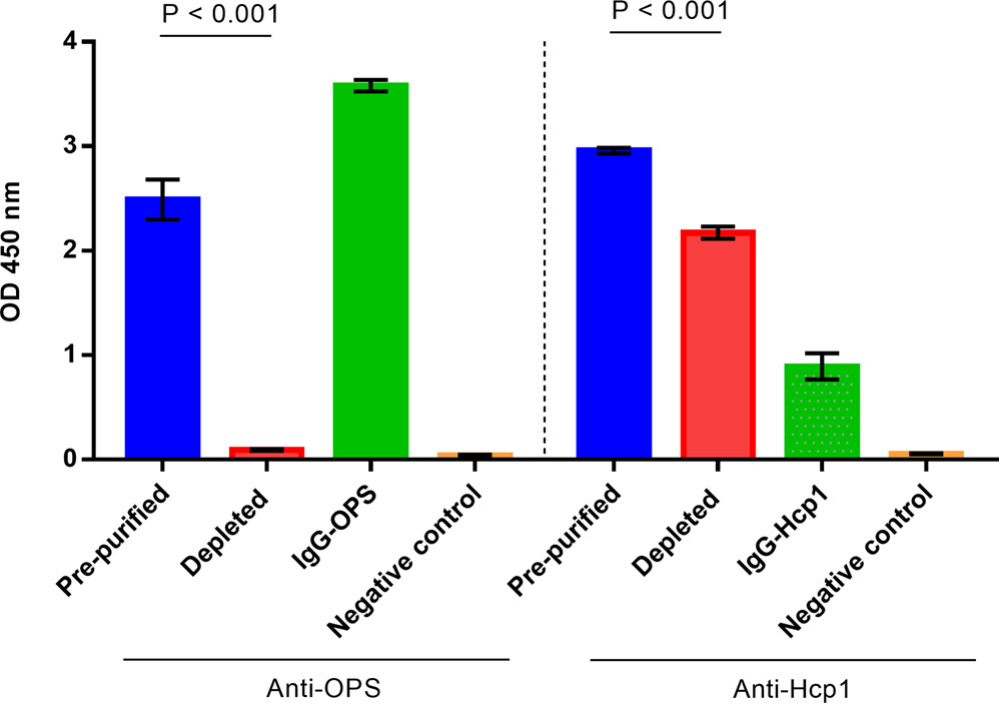
Antibody activity of prepurified pooled serum, depleted serum, purified IgG anti-OPS (IgG-OPS) and purified IgG anti-Hcp1 (IgG-Hcp1) against OPS or Hcp1 antigen. The antibody activity was determined by ELISA using serum concentration at 1 μg/mL and antigen concentration at 2.5 μg/mL for Hcp1 and 1.0 μg/mL for OPS. Negative control was pooled serum samples from healthy donors in non-area of endemicity. Two independent experiments were performed.

We next evaluated the reactivity of the various pooled serum samples and purified IgG fractions by ELISA. To facilitate these studies, all samples were adjusted to 1 μg/mL of protein. As expected, analysis of the prepurified serum samples demonstrated that they had strong reactivity with OPS or Hcp1 while noticeable decreases in reactivity were observed for their paired depleted samples (Fig. 3). Interestingly, depletion of total Ig-OPS from its paired prepurified sample appeared to be more efficient than depletion of total Ig-Hcp1 from its paired prepurified sample with the OPS depleted sample reaching reactivity levels similar to the negative control. Importantly, however, these analyses indicated that we were successful in isolating highly purified OPS- and Hcp1-reactive IgG for use functional assays.

### IgG-OPS and IgG-Hcp1 antibodies enhance ADCP activities.

**FIG 4:**
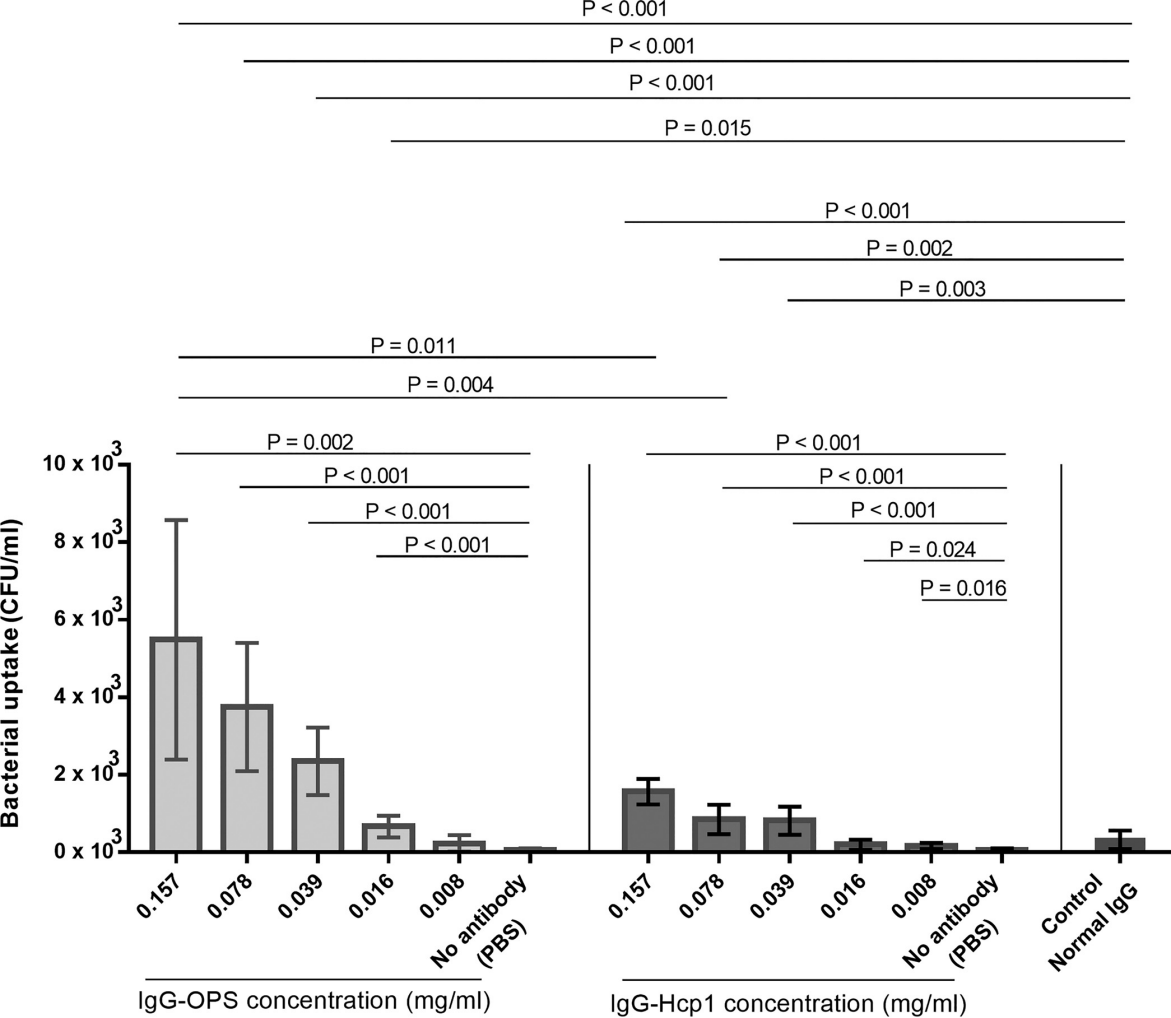
Antibody-dependent cellular phagocytosis (ADCP) activity of purified IgG anti-OPS (IgG-OPS) and IgG anti-Hcp1 (IgG-Hcp1) at different concentrations. PBS and IgG from healthy donors (normal IgG) were controls. The ADCP activity was determined by colony count to enumerate live bacteria in THP-1 cells. Three-independent experiments were performed.

To assess the functional activity of IgG-OPS and IgG-Hcp1, we used human leukemic monocyte cell line THP-1 to conduct ADCP assays. Results of these studies demonstrated significantly higher opsonizing activity associated with IgG-OPS compared to PBS and the IgG control at all concentrations tested except for 0.008 mg/mL (Fig. 4). ADCP testing of IgG-Hcp1 also showed significantly higher activity than PBS and the IgG control but lower activity than IgG-OPS (Fig. 4) for IgG-Hcp1 concentrations of 0.157 and 0.078 mg/mL. IgG-OPS antibodies at concentrations of 0.157 and 0.078 mg/mL enhanced bacterial uptake by approximately 3 times compared to the IgG-Hcp1 antibody at the same concentrations (*P* = 0.026 and *P* = 0.002, respectively). We did not observe the difference in live/dead cells among THP1 cells incubated with various IgG concentrations and PBS control. These results suggest IgG-OPS and IgG-Hcp1 enhance ADCP activities in human monocytic cells.

### IgG-OPS enhances C3b deposition on B. pseudomallei K96243.

**FIG 5:**
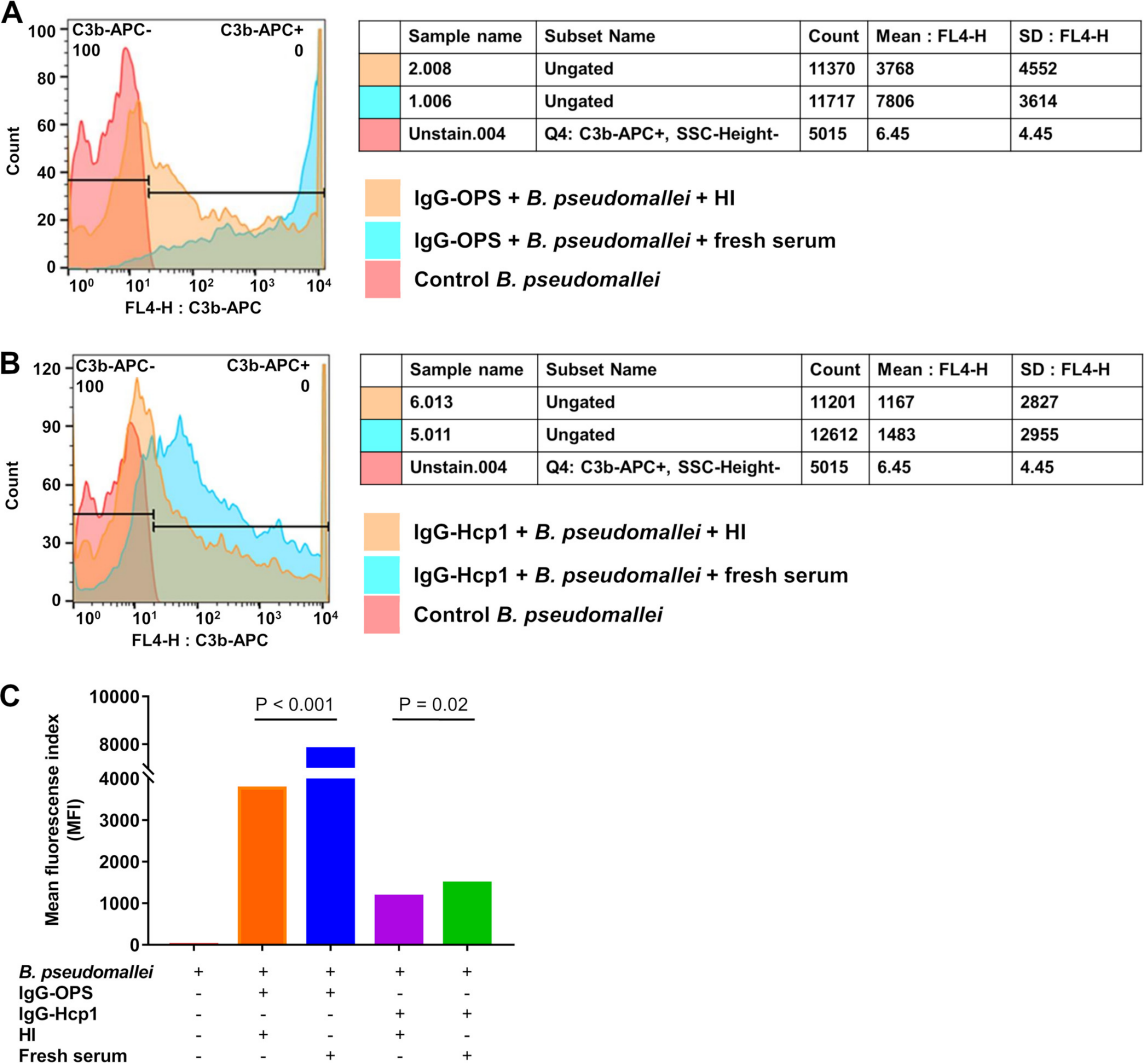
Antibody-dependent complement deposition (ADCD) of B. pseudomallei K96243 surface opsonized with IgG anti-OPS (IgG-OPS) and IgG anti-Hcp1 (IgG-Hcp1). ADCD was assessed by the measurement of complement component C3b on the surface of target cells. A fresh healthy donor serum sample was used as a complement source. Negative controls were heat-inactivated serum sample (HI) (no complement) and unopsonized B. pseudomallei K96243. C3b deposition on bacterial cells was examined by probing with allophycocyanin (APC) conjugated anti-human C3b. Complement deposition was detected using flow cytometry and data are shown as histograms and mean fluorescence index (MFI). (A) Deposition of C3b complement component on B. pseudomallei K96243 opsonized with IgG-OPS. (B) Deposition of C3b complement component on B. pseudomallei K96243 opsonized with IgG-Hcp1. (C) Data are presented as mean fluorescence index (MFI). Three-independent experiments were performed.

To study antibody function in enhancing complement deposition on the bacterial surface, C3b deposition was assessed by flow cytometry and is represented as mean fluorescent index (MFI) (Fig. 5). MFI of B. pseudomallei and IgG-OPS or IgG-Hcp1 incubated with fresh healthy donor serum (fresh serum) as a source of complement was compared with bacteria incubated with heat-inactivated serum (HI) or PBS as controls. Results demonstrated significantly higher C3b deposition on B. pseudomallei K96243 incubated with IgG-OPS and fresh serum (MFI, 7806), compared with the incubation of bacteria with HI (MFI, 3768) or PBS (MFI, 6.45) (fresh serum versus HI, *P* < 0.001) (Fig. 5A and C). A similar pattern, but considerably lower MFI, was observed with B. pseudomallei treated with IgG-Hcp1 and fresh serum. The MFI of K96243 and IgG-Hcp1 incubated with fresh serum, HI, and the PBS control were 1483, 1167 and 6.45, respectively (fresh serum versus HI, *P* = 0.02) (Fig. 5B and C). Collectively, these results demonstrate that IgG-OPS is superior to IgG-Hcp1 in promoting the deposition of C3b on the surface of B. pseudomallei.

## DISCUSSION

Currently, there is no vaccine for melioidosis. Understanding humoral immune responses against different B. pseudomallei antigens is important for vaccine development. In this study, we purified and characterized the functional activities of specific antibodies against two vaccine candidate antigens, OPS and Hcp1, using pooled serum samples from acute melioidosis patients and healthy donors living in Northeast Thailand (area of endemicity) and in Bangkok (non-area of endemicity). We observed that the ADCP activity of pooled serum from acute melioidosis patients for B. pseudomallei were significantly higher than the activity of serum obtained from healthy donors. We successfully purified specific IgG antibodies against OPS and Hcp1 antigens from acute melioidosis patient serum and healthy donors. In human melioidosis, we observed that ADCP activity of both antigen-specific IgGs were correlated with concentrations in dose-dependent manner. We also showed that the functional activities of purified IgG-OPS from melioidosis patient serum was significantly greater than the activity of IgG-Hcp1.

Our ADCP results in this study confirmed findings from a previous report by Chaichana et al. that serum from melioidosis patients can enhance opsonization of B. pseudomallei in a THP-1 cell derived macrophage model (25). Although a follow-up study by Chaichana et al. demonstrated that high levels of total IgG2 and the expression of FcγRIIa-H/R131, an intermediate-affinity IgG2 receptor, were associated with protection against death in acute melioidosis (26), they did not identify the specific target antigen for IgG2 as they used whole cells of B. pseudomallei in their assay. Herein, we purified IgG against OPS and Hcp1 antigens and demonstrated that these specific antibodies promoted phagocytosis and complement fixation. The results of this study suggest that IgG-OPS enhanced phagocytosis of and C3b deposition on B. pseudomallei K96243 compared to IgG-Hcp1. This might be explained by the nature of polysaccharide (OPS) versus protein antigen (Hcp1) that induced different subclasses of IgG antibodies. Indeed, our previous study showed that IgG1 and IgG2 were major subclasses of IgG antibody against OPS whereas IgG1 was a major IgG subclass for Hcp1 in serum samples from acute melioidosis patients (17). It is possible that these antibodies enhance bacterial internalization and cells-to-cells spreading but the bacteria were not killed by the host cells due to the inability of B. pseudomallei-infected macrophages to produce IFN-γ and IFN-β. However, a study has demonstrated that stimulation of infected macrophages with either of these interferons can result in increased iNOS expression and reduced intracellular survival of B. pseudomallei (27).

ADCD can be associated with both classical and alternative complement pathways. It has been previously reported that the capsule of B. pseudomallei can prevent C3b deposition on the surface of bacteria (28). However, our study indicates an important role for IgG-OPS in C3b deposition on B. pseudomallei and suggests that high levels of antibodies may play a role in controlling B. pseudomallei infections. The role of specific antibodies for complement activation against other bacterial and viral infections have been demonstrated previously, for example, in Neisseria meningitidis serogroup B (29), Escherichia coli 0111B4 (30), Salmonella Typhi (31), immunodeficiency virus (HIV) antibodies (32). These studies show human monoclonal antibodies to specific antigens are most efficient in cell lysis and C3 deposition on infected cells (32). However, we did not measure the role of antibodies beyond the C3b deposition, and this requires further study.

Several studies have demonstrated the protective capacity of OPS-specific monoclonal and polyclonal antibodies in animal models of melioidosis (20, 33, 34). Studies have also shown that antibodies against B. pseudomallei LPS II (also known as type A LPS) are significantly higher in patients who survived than in those who died as well as in patients with nonsepticemic versus septicemic melioidosis (24). Furthermore, studies have described correlations between OPS- and Hcp1-specific IgG subclasses and improved outcomes for melioidosis patients (11, 18). Our current study represents the first of its kind to demonstrate specific functional activities associated with affinity-purified human OPS- and Hcp1-specific antibodies which helps to better establish correlates of antigen-induced immunity against B. pseudomallei. Thus, this study provides valuable insights toward the development of OPS- and Hcp1-based vaccines to combat melioidosis.

There were some limitations to this study. We did not use fresh serum samples from melioidosis patients therefore antibody functions may be partly degraded during long-term storage. It is known that a number of healthy people who live in areas of endemicity have high antibody levels against B. pseudomallei culture filtrate antigen (CFA) (35, 36). This study did not test the differences in functional activity of specific antibodies due to the limited amounts of serum samples. Further studies are required to investigate the potential role(s) of antibodies against OPS and Hcp1 in healthy individuals.

In conclusion, the results of this study confirm that melioidosis patients have high levels of antibodies to OPS and Hcp1 and suggest the functional roles of purified human IgG specific for these antigens enhances bacterial uptake into human monocytic cells and C3b deposition on the B. pseudomallei K96243 cell surface. These results provide insights into the functional activities of antibodies against these two potential vaccine candidates and supports using OPS and Hcp1 antigens as targets for vaccine development.

## MATERIALS AND METHODS

### Serum preparation.

Left-over acute and convalescent-phase sera from 100 melioidosis patients who were culture positive for B. pseudomallei were pooled and used for purification of OPS- or Hcp1-specific antibodies. A total of 400 mL of pooled serum was obtained from melioidosis patients. A total of 3.5 mL of pooled serum from 7 healthy donors in Udon Thani in northeast Thailand (an area of endemicity of melioidosis) and 2.5 mL of pooled serum from 5 healthy donors in Bangkok (non-area of endemicity of melioidosis) were used as controls. The serum samples were passed through gauze and then filtered through 0.20 μm filters (Acrodisc, PR, USA).

### Coupling of B. pseudomallei OPS to UltraLink Hydrazide resin.

To facilitate purification of specific antibodies against B. pseudomallei OPS, type A LPS was extracted from the select agent excluded strain B. pseudomallei RR2808 using a modified hot phenol method (37, 38). Purified OPS antigens were then obtained by acid hydrolysis and size exclusion chromatography essentially as previously described (37, 39). The OPS was then oxidized with sodium metaperiodate as previously described (37) and conjugated to UltraLink Hydrazide resin (Thermo Scientific, USA) via reductive amination following the manufacturer’s instructions. The resulting OPS-coupled resin was stored at 4°C until use.

### Coupling of Hcp1 to UltraLink Biosupport resin.

We have previously reported that B. pseudomallei Hcp1 and B. mallei Hcp1 are 99.4% identical at the amino acid level (e.g., they differ by only one amino acid; [17]). Furthermore, studies in our lab have demonstrated that B. pseudomallei Hcp1 and B. mallei Hcp1 can be used interchangeably as ELISA coating antigens and that human immune serum samples react similarly with both proteins. To facilitate the purification of Hcp1-specific antibodies, recombinant B. mallei Hcp1 was purified from E. coli as previously described (17). The purified Hcp1 was then coupled to UltraLink Biosupport resin (Thermo Scientific, USA) following the manufacturer’s instructions. The resulting Hcp1-coupled resin was stored at 4°C until use.

### Purification of OPS- and Hcp1-specific antibodies from pooled human melioidosis serum.

To purify OPS- or Hcp1-specific antibodies from human immune serum, the pooled serum was diluted 1:1 with binding buffer, 0.5× BupH PBS pH 7.2 (Thermo Scientific, USA) for anti-OPS and 0.6 M sodium citrate and 0.1 M MOPH (pH 7.5) for anti-Hcp1. The buffered serum was then passed over 1 mL of the OPS or Hcp1 coated beads which were preequilibrated with binding buffer, washed with 15 resin volumes of binding buffer following which the antibodies were stripped from the resin with elution buffer, 100 mM glycine-HCl (pH 2.5) for anti-OPS and 100 mM glycine, 2% acetic acid (pH 2.2) for anti-Hcp1. The eluate was collected into a 1/10 volume of 1 M Tris pH 9.0. The sample was then dialyzed against multiple changes of 0.5× BupH^T^ PBS for anti-OPS and 20 mM Sodium phosphate, pH 7.0 for anti-Hcp1. Precipitates were removed by centrifugation and the filter sterilized material was passed over Protein G Hi-Trap columns (GE Healthcare Bio-Sciences, Sweden) to isolate purified IgG.

### Purification of IgG-OPS and IgG-Hcp1.

Protein G columns (GE Healthcare Bio-Sciences, Sweden) were used to purify IgG-OPS or IgG-Hcp1 from total anti-OPS or total anti-Hcp1 antibodies. Collection tubes were prepared by adding 60 μL of 1 M Tris-HCl, pH 9.0 per mL for each fraction to be collected. The columns were washed with 10 column volumes of binding buffer, 20 mM Sodium phosphate (pH 7.0) at 1 mL/min. A total of 5 mL sample was applied to the columns followed by washing with 10 mL of binding buffer. The flow through from the samples (anti-OPS IgG or anti-Hcp1 IgG depleted samples) was then collected. IgG-OPS or IgG-Hcp1 were eluted with 10 column volumes of elution buffer, 0.1 M Glycine-HCl (pH 2.7) and collected as 10 individual fractions. The purified fractions were buffered exchanged by dialysis against PBS. Normal human serum IgG used as IgG controls were prepared from 3.5 mL pooled serum samples from 7 healthy donors from Northeast Thailand and 5 healthy donors from Bangkok Thailand. The protein concentrations were determined using a BCA protein assay kit (Pierce, Thermo Fisher Scientific, USA). The yield of IgG was 7 mL with concentration of 3.947 mg/mL. The protein purity was determined by SDS-PAGE and Coomassie blue staining as previously described by Laemmli et al. (40). SDS-PAGE was performed with 14% gel.

### Determination of antibody activity.

The reactivity of serum and IgG against OPS and Hcp1 antigens was determined by Enzyme-linked Immunosorbent assay (ELISA) as previously described (16, 17). Samples were diluted in assay diluent and assays performed in duplicate. Briefly, 50 μL of 2.5 μg/mL of Hcp1 or 1.0 μg/mL of OPS was coated on 96 microwell plate (Nunc, Sigma-Aldrich, Germany) at 4°C overnight. The plate was washed with PBS containing 0.05% Tween, then blocked with skim milk at 37°C for 2 h. 50 μL of the concentration of 1 μg/mL of prepurified serum, IgG-OPS, IgG-Hcp1, depleted serum, and negative control (pooled serum from healthy donors) were added and the plate was incubated at RT for 30 min. After washing with PBS, 50 μL of 1:2000 dilution of HRP-conjugated rabbit anti-human IgG (Dako) was added and incubated at RT for 30 min. The plate was washed and the color was developed using 50 μL of 3,3′,5,5′ tetramethylbenzidine (TMB) with peroxidase (Novex, Life Technologies, MD, USA). The reaction was quenched using 1N HCl. The absorbance was measured at an optical density (OD) of 450 nm using microplate reader (Sunrise, Tecan, Switzerland).

### Preparation of human monocytic cell line THP-1.

The opsonizing activities of purified IgG-OPS and IgG-Hcp1 antibodies from melioidosis patients were determined using the human monocytic cell line THP-1 (ATCC TIB 202). The THP-1 cells were cultivated in Roswell Park Memorial Institute (RPMI) 1640 Medium (Gibco, MS, SA) with 10% fetal bovine serum (10% FBS) and incubated at 37°C with 5% CO_2_ for 2 days prior to use. Cells were harvested, washed twice with RPMI by centrifugation at 200 × *g* for 5 min, and counted. The cells were diluted in RPMI to obtain a concentration of 1× 10^6^ cells/mL and incubated at 37°C with 5% CO_2_ before use in ADCP assays.

### Bacterial culture and opsonization.

To prepare bacterial inoculums, B. pseudomallei strain K96243 from a frozen stock was cultured on LB agar and incubated at 37°C overnight. Isolated colonies were picked, inoculated in 20 mL of LB broth and incubated with shaking at 37°C, 200 rpm overnight. The bacteria were diluted with LB broth and adjusted to OD600 nm = 0.18 to obtain a concentration of 5× 10^6^ CFU/mL. All experiments involving B. pseudomallei were performed in a biosafety level 3 laboratory.

Purified antibodies (IgG-OPS and IgG-Hcp1) were diluted with sterile PBS to concentrations of 0.157, 0.078, 0.039, 0.016, and 0.008 mg/mL. Purified IgG controls from healthy donors were diluted in PBS to 0.157 mg/mL. Pooled serum samples were diluted with RPMI at 1:5 before use.

### Antibody-dependent cellular phagocytosis (ADCP) assay.

ADCP assays were performed using THP-1 cells in suspension and centrifuged during the washing steps at multiplicity of infection (MOI) of 5 bacteria per cell (CFU/cell). A total of 200 μL of B. pseudomallei K96243 at 1× 10^6^ CFU/mL were incubated with 20 μL of diluted antibody in PBS or diluted pooled serum in RPMI (5 μL serum in 45 μL RPMI) in a 1.5-mL microtube (Axygen, Corning, USA) at 37°C for 1 h. The opsonized bacteria were then washed with RPMI twice by centrifugation at 4,000 × *g* for 15 min. The pellet was resuspended with 220 μL of RPMI, then transferred into 96-well U bottom plate with THP-1 cells and incubated at 37°C with 5% CO_2_ for 2 h. Each sample was performed in triplicate (25, 26).

Opsonized B. pseudomallei K96243 was prepared as described above and incubated with THP-1 cells for 2 h. Extracelllular bacteria were removed by washing with RPMI 3 times. THP-1 cells were treated with 500 μg/mL of kanamycin for 2 h. Live and dead THP1 cells were examined using an inverted light microscope. The cells were washed 3 times with PBS and 1 mL of 0.1% Triton X-100 (Thermo Fisher Scientific, USA) in PBS was added and incubated at RT for 10 min. Cell lysates were diluted in PBS and 10 μL was dropped on LB agar. The plates were incubated at 37°C for 24–48 h to determine colony counts (41).

### Antibody-dependent complement deposition (ADCD) assay.

ADCD was assessed by measuring deposition of complement component C3b on the surface of target cells as previously described (42). B. pseudomallei K96243 was incubated with 0.784 μg/mL of IgG-OPS or IgG-Hcp1 antibodies at 37°C for 30 min. The opsonized bacteria were washed with PBS. A fresh healthy donor serum sample diluted in veronal buffer (1:10 dilution) was added as a complement source and incubated at 37°C for 30 min, then washed with 15 mM EDTA in PBS. Heat-inactivated serum (56°C for 30 min) was used as a negative control. C3b deposition on bacterial cells was probed with allophycocyanin (APC) conjugated anti-human C3b, clone: 1H8 (Mouse IgG2a) (Cedarlane, CA) at RT for 15 min in the dark and fixed with 4% paraformaldehyde. Complement deposition was detected using flow cytometry (FACSCalibur flow cytometer, Becton, Dickinson, CA). ADCD assays were performed in triplicate. Data were analyzed and shown as histograms and mean fluorescence index (MFI).

### Statistical analysis.

The data were analyzed using GraphPad Prism software version 7.0 (GraphPad Software Inc, La Jolla, CA). The data are presented as bar graphs with mean ± standard deviation (SD). Student's *t* test was used to compare the quantitative data between two groups. One-way ANOVA were used to compare the quantitative data of three or more groups. Two or three independent experiments were performed. A statistically significant difference was *P* value < 0.05.

### Ethics statement.

This study was approved by the ethics committee of Faculty of Tropical Medicine, Mahidol University (MUTM 2019-035-03) and the biosafety committee of the Faculty of Tropical Medicine, Mahidol University (MU 2021-010). The study was conducted under the principles of Declaration of Helsinki (2008) and Good Clinical Practice (GCP) guidelines. Written informed consent was obtained from all participants or their relatives.
